# Supplementary material for: Content-rich biological network constructed by mining PubMed abstracts
Source: BMC Bioinformatics. 2004 Oct 8;5:147. doi: 10.1186/1471-2105-5-147 (PMC528731; doi:10.1186/1471-2105-5-147)
Supplement: Additional File 2 — The original results of the above study (non-essential files are deleted to keep the file size under the limit set by BMC bioinformatics). [file 1471-2105-5-147-S2.bz2 › chilibotAdditionalFile2/dip05/765ID9732274E18154/html/PNP_RHLB.html]

 


 **PNP** and **RHLB** 
  
Found 13 abstracts in PubMed, retrieved 05.  
 

 What does Google say? 
 PDF only 
| .edu only 

---

**Interactive relationship** (e.g. stimulation, inhibition, etc)

**Neutral relationship**- BIAcore analysis revealed real time, direct binding for bimolecular interactions between Rh1B units and for the  **RhlB**  interaction with PNPase  [ **PNP** ] .  Ref: 12181321 J Biol Chem, 2002
- Earlier work has shown that the C terminal region of RNase E is a scaffold for the binding of degradosome components and has identified specific RNase E segments necessary for its interaction with polynucleotide phosphorylase  [ **PNP** ]  PNPase  [ **PNP** ] ,  **RhlB**  RNA helicase, and enolase.  Ref: 11134527 Proc Natl Acad Sci U S A, 2001
- DEAD box  **RhlB**  RNA helicase physically associates with exoribonuclease PNPase  [ **PNP** ]  to degrade double stranded RNA independent of the degradosome assembling region of RNase E.  Ref: 12181321 J Biol Chem, 2002
- RNase E is a large multidomain protein with N terminal ribonucleolytic activity, an RNA binding domain and a C terminal scaffold that binds PNPase  [ **PNP** ] , enolase and  **RhlB** .  Ref: 12035760 Biochem Soc Trans, 2002
- **RhlB**  in vitro can facilitate the degradation of structured RNA by PNPase  [ **PNP** ] .  Ref: 12035760 Biochem Soc Trans, 2002

**Non-interactive relationship** (e.g. studied together, co-existance, homology, etc.)

- The most important recent developments have been the discovery that the degradosome a multiprotein complex containing an endoribonuclease RNase E, an exoribonuclease polynucleotide phosphorylase  [ **PNP** ] , and a DEAD box helicase  **RhlB**  has a central role in mRNA degradation.  Ref: 10684583 Bioessays, 2000
- Furthermore, in the absence of RNase E, purified  **RhlB**  can carry out ATP dependent unwinding of double stranded RNA and consequently modulate degradation of double stranded RNA together with the exonuclease activity of PNPase  [ **PNP** ] .  Ref: 12181321 J Biol Chem, 2002
- Using an E. coli two hybrid system, together with BIAcore apparatus, we investigated the ability of three proteins, polynucleotide phosphorylase  [ **PNP** ]  PNPase  [ **PNP** ] ,  **RhlB**  RNA helicase, and enolase, a glycolytic protein, to interact physically and functionally independently of RNase E.  Ref: 12181321 J Biol Chem, 2002
- However, binding of  **RhlB**  or PNPase  [ **PNP** ]  to enolase was not detected under the same conditions.  Ref: 12181321 J Biol Chem, 2002
- Two other components are a 3 exoribonuclease polynucleotide phosphorylase  [ **PNP** ] , PNPase  [ **PNP** ]  and a DEAD box RNA helicase RNA helicase B,  **RhlB** .  Ref: 12035760 Biochem Soc Trans, 2002
- These results provide evidence for the first time that both functional and physical interactions of individual degradosome protein components can occur in the absence of RNase E and raise the prospect that the RNase E independent complexes of  **RhlB**  RNA helicase and PNPase  [ **PNP** ] , detected in vivo, may constitute mini machines that assist in the degradation of duplex RNA in structures physically distinct from multicomponent RNA degradosomes.  Ref: 12181321 J Biol Chem, 2002
